# ASRmiRNA: Abiotic Stress-Responsive miRNA Prediction in Plants by Using Machine Learning Algorithms with Pseudo *K*-Tuple Nucleotide Compositional Features

**DOI:** 10.3390/ijms23031612

**Published:** 2022-01-30

**Authors:** Prabina Kumar Meher, Shbana Begam, Tanmaya Kumar Sahu, Ajit Gupta, Anuj Kumar, Upendra Kumar, Atmakuri Ramakrishna Rao, Krishna Pal Singh, Om Parkash Dhankher

**Affiliations:** 1 ICAR-Indian Agricultural Statistics Research Institute, Indian Council of Agricultural Research, New Delhi 110012, India; ajit@icar.gov.in (A.G.); anujbioinfo91@gmail.com (A.K.); 2 ICAR-National Institute for Plant Biotechnology, Indian Council of Agricultural Research, New Delhi 110012, India; shaba.shb@gmail.com; 3 ICAR-National Bureau of Plant Genetic Resources, Indian Council of Agricultural Research, New Delhi 110012, India; tanmayabioinfo@gmail.com; 4 Department of Molecular Biology, Biotechnology and Bioinformatics, College of Basic Sciences and Humanities, CCS Haryana Agricultural University, Hisar 125004, India; baliyan.upendra@gmail.com; 5 Indian Council of Agricultural Research (ICAR), New Delhi 110012, India; rao.cshl.work@gmail.com; 6 Biophysics Unit, College of Basic Sciences and Humanities, GB Pant University of Agriculture & Technology, Pantnagar 263145, India; kps_biophysics@yahoo.co.in; 7 Mahatma Jyotiba Phule Rohilkhand University, Bareilly 243005, India; 8 Stockbridge School of Agriculture, University of Massachusetts Amherst, Amherst, MA 01003, USA

**Keywords:** abiotic stress, miRNAs, stress-responsive genes, machine learning, computational biology

## Abstract

MicroRNAs (miRNAs) play a significant role in plant response to different abiotic stresses. Thus, identification of abiotic stress-responsive miRNAs holds immense importance in crop breeding programmes to develop cultivars resistant to abiotic stresses. In this study, we developed a machine learning-based computational method for prediction of miRNAs associated with abiotic stresses. Three types of datasets were used for prediction, i.e., miRNA, Pre-miRNA, and Pre-miRNA + miRNA. The pseudo *K*-tuple nucleotide compositional features were generated for each sequence to transform the sequence data into numeric feature vectors. Support vector machine (SVM) was employed for prediction. The area under receiver operating characteristics curve (auROC) of 70.21, 69.71, 77.94 and area under precision-recall curve (auPRC) of 69.96, 65.64, 77.32 percentages were obtained for miRNA, Pre-miRNA, and Pre-miRNA + miRNA datasets, respectively. Overall prediction accuracies for the independent test set were 62.33, 64.85, 69.21 percentages, respectively, for the three datasets. The SVM also achieved higher accuracy than other learning methods such as random forest, extreme gradient boosting, and adaptive boosting. To implement our method with ease, an online prediction server “ASRmiRNA” has been developed. The proposed approach is believed to supplement the existing effort for identification of abiotic stress-responsive miRNAs and Pre-miRNAs.

## 1. Introduction

MicroRNAs (miRNAs) are 20–24 nucleotides long, small non-coding RNA molecules, widely distributed in the plant kingdom [1,2]. By regulating the expression of stress-responsive genes, miRNAs play a vital role in plant response to different abiotic stresses and are thus regarded as the bio-regulators of plant stress response [3,4,5,6]. Due to the importance of miRNAs in regulating the plant response to different environmental stresses, many experimental and computational studies have been carried out to identify and characterize abiotic stress-responsive miRNAs. Specifically, miRNAs in response to drought [7,8,9,10,11,12,13,14,15], cold [16,17,18,19], heat [20,21,22], light [23,24,25,26,27,28,29,30], salt [15,31,32], and oxidative [33,34,35,36,37,38] stresses have been identified in several crop species. In addition, miRNAs involved in mineral-nutrient and mechanical stresses have also been reported in previous studies [39,40,41].

The miRNAs response to abiotic stresses depends upon the types of genotype, stress, tissue, and miRNA [42]. Down-regulated expression of miR408 in rice [11], cotton [43], and peach [44] and up-regulated expression miR408 in *Arabidopsis* [8], *Medicago* [45], and barley [13] during drought stress is an example of genotype-dependent response of miRNAs. With regard to tissue-dependent response of miRNAs, the study by Wang et al. [46] revealed the altered expression profile of miRNAs in roots as compared to leaves in response to drought and salinity stresses in cotton. The miR169 was inhibited by drought stress [47] but was found to be induced by salinity treatment in *Arabidopsis* [48], which demonstrates that abiotic stresses induce the expression of miRNAs in a stress-dependent manner. Similarly, miR398 was induced by UVB light in *Arabidopsis* but was inhibited by salinity, cold, and oxidative stress [34,49]. As far as plant response to abiotic stresses in miRNAs-dependent manner is concerned, the expression of miR397 was significantly induced but the expression of miR398 was significantly inhibited in *Arabidopsis* under salinity stress [8]. All the above cited studies suggest that miRNAs play a significant role in plant response to different abiotic stresses.

Most of the abiotic stress-responsive miRNAs have been identified by experimental methods such as RT-PCR, cloning, RNA-microarrays, and northern blots [50,51]. In addition, the NGS and deep sequencing technologies have also led to the identification of a large number of abiotic stress-responsive miRNAs [52]. All the miRNAs identified through wet lab experiments and sequencing methods have been populated in the form of databases such as miRBase [53], miRNEST [54], PMRD [55], miRPlant [56], PlantMirnaT [57], PASmiR [58], PncStress [59], NtUE-Webresource [41], and others [50]. The PncStress database is the most updated, and it contains the experimentally validated miRNA sequences associated with different environmental stresses. Nevertheless, the experimental methods and high throughput sequencing technologies are resource intensive, as far as identification of stress-responsive miRNAs is concerned. Further, no computational tool is available for the prediction of abiotic stress-responsive miRNAs using the miRNA sequence data. Keeping in view the importance of miRNAs in plant response to abiotic stresses and non-availability of computational methods for predicting such miRNAs, our objective in this study is to develop a machine learning-based computational tool for predicting abiotic stress-responsive miRNAs using the features derived from the miRNA sequences. The present study is expected to supplement the wet-lab experiments and other sequencing technologies for identification of miRNAs under abiotic stresses.

## 2. Results

### 2.1. Feature Selection Analysis

**Figure 1:**
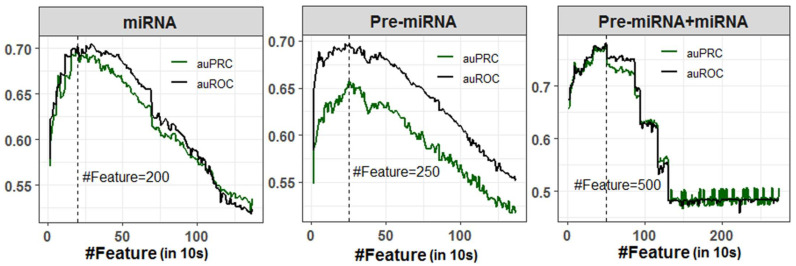
Feature selection for miRNA, Pre-miRNA, and Pre-miRNA + miRNA datasets. The optimal number of features were selected based on the higher accuracies in terms of auROC and auPRC. A total of 200, 250, and 500 features were selected for miRNA, Pre-miRNA, and Pre-miRNA + miRNA datasets, respectively.

A total of 1372 numeric features (Pseudo *K*-tuple nucleotide composition: PseKNC) was generated for each miRNA and Pre-miRNA sequence. However, the features were highly correlated as there were large numbers of sparse features generated due to the smaller sequence length of miRNAs (20–24 nucleotides) and Pre-miRNAs (80–120 nucleotides). Correlated or redundant features may negatively affect the classification accuracy. Thus, SVM-RFE feature selection method was employed to select important features for the classification of stress-responsive miRNAs and non-stress-responsive miRNAs with higher accuracy, where randomly selected 50% observations of the dataset were utilized for classification. An optimal feature set containing 200 features was selected for classification using miRNA sequences, where the values of auROC (70.19%) and auPRC (70.11%) were observed to be higher (Figure 1). While classification was performed with Pre-miRNA dataset, higher auROC (69.74%) and auPRC (65.73%) were obtained with an optimal subset of 250 selected features (Figure 1). As far as classification with Pre-miRNA + miRNA dataset is concerned, higher classification accuracies (auROC: 78.04% and auPRC: 77.08%) were found with 500 selected features (Figure 1). It was also found that the classification accuracies (auROC and auPRC) were increased up to 200, 250, and 500 selected features, respectively, for miRNA, Pre-miRNA, and Pre-miRNA + miRNA datasets and started declining thereafter (Figure 1). Lowest accuracies were observed for all three datasets when classification was performed using all the PseKNC features.

### 2.2. Cross-Validated Prediction Analysis with Selected Features

Final prediction analysis was performed for miRNA, Pre-miRNA and Pre-miRNA + miRNA datasets with the respective selected feature sets, where the SVM with RBF kernel was utilized as predictor. Moreover, to achieve maximum classification accuracy, the parameters gamma (γ) and cost (C) were optimized through a grid search approach with $\gamma =2^{-5}:2^{5}$ and $C=2^{-5}:2^{5}$, with step size 2. However, the higher accuracies were found with the default parameters (γ=1/#Feature, C=1) for all three datasets and are given in Table 1. 

The performance metrics were observed to be ~64–70% with the miRNA dataset, whereas for the Pre-miRNA dataset the performance metrics were found be ~63–69%. As far as prediction with Pre-miRNA + miRNA dataset is concerned, the prediction accuracies such as Sen (74.00%), Spe (68.80%), Acc (71.40%), Pre (70.34%), F-score (72.12%), auROC (77.94%), and auPRC (77.32%) were observed to be higher than the respective accuracies obtained with miRNA and Pre-miRNA datasets. In contrast, there was not much difference observed between the accuracies of miRNA and Pre-miRNA datasets. It was also observed that the sensitivities were higher than the corresponding specificities for all three datasets (Table 1). The higher accuracy of prediction with Pre-miRNA + miRNA dataset may be attributed to the use of more features (550) as compared to those of miRNA (200) and Pre-miRNA (250) datasets.

### 2.3. Performance Analysis with Other Learning Methods

The performance of predicting abiotic stress-responsive miRNAs was further analyzed with other start-of-the-art machine learning algorithms such as random forest (RF; Breiman [60]), extreme gradient boosting (XGB; Chen and Guestrin [61]), and adaptive boosting (ADB; Freund and Schapire [62]). The R-packages *randomForest* [63], *xgboost* [64], and *adabag* [65] were, respectively, utilized for implementing the RF, XGB, and ADB learning methods. For prediction with the miRNA dataset, the accuracies of RF (Sen: 55.20%, Spe: 58.13%, Acc: 56.66%, Pre: 56.86%, F-score: 56.02%, auROC: 58.88%, auPRC: 69.96%) were observed to be higher than those of XGB (51.21%, 56.00%, 53.61%, 53.78%, 52.46%, 54.79%, 56.03%) and ADB (52.26%, 57.06%, 54.67%, 54.91%, 53.55%, 57.45%, 57.01%) (Table 2). 

Similarly, the performance metrics of RF (58–65%) with the Pre-miRNA dataset were further observed to be 2–10% higher than those of XGB (54–58%) and 2–7% higher than those of ADB (57–60%), with the exception of specificity (Table 2). When predicting with the Pre-miRNA + miRNA dataset, the prediction accuracies of RF were also observed higher as compared to those of the XGB and ADB learning methods. In addition, the differences in accuracies between RF and XGB and between RF and ADB were less than those of prediction with miRNA and Pre-miRNA datasets (Table 2). Similar to SVM, the performance of RF, XGB, and ADB were observed to be higher for the Pre-miRNA + miRNA dataset as compared to the miRNA and Pre-miRNA datasets. Further, the performance accuracies of XGB were observed to be higher than those of ADB. The prediction accuracies of XGB and ADB methods were found less than 60% with Pre-miRNA and miRNA datasets, and more than 60% with the Pre-miRNA + miRNA dataset (Table 2). 

### 2.4. Comparison of SVM with Other Learning Methods

**Figure 2:**
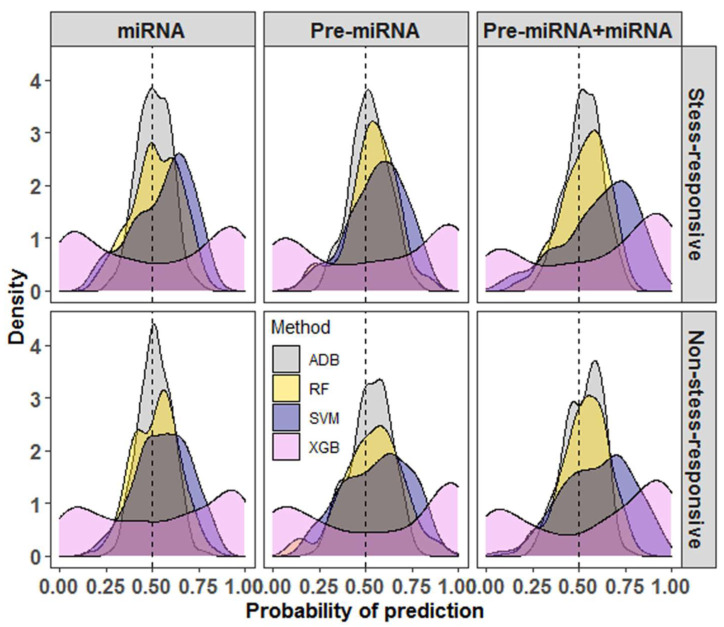
Density graphs for the probabilities of prediction for different machine learning methods. It can be seen that most of the probabilities of prediction with SVM are higher than the random guess (0.5) as compared to random forest (RF), extreme gradient boosting (XGB), and adaptive boosting (ADB) methods. The XGB is observed to be the lowest performer among the considered methods. The variability in the prediction probabilities is lowest for the ADB and highest for the XGB methods.

When the performance of SVM was compared with that of other learning algorithms, SVM was found to outperform RF, XGB, and ADB in all three datasets (Table 1 and Table 2). For Pre-miRNA and miRNA datasets, the accuracies of SVM were >60%, whereas the accuracies were less than 60% for RF, XGB, and ADB methods. Similarly, for the Pre-miRNA + miRNA dataset, SVM achieved more than 70% accuracy as compared to less than 70% accuracy for the learning methods RF, XGB, and ADB (Table 1 and Table 2). One of the probable reasons for the higher accuracy with SVM is the features selected through SVM-RFE may not be an optimal feature set for other learning methods. It was also found that most of the prediction probabilities of RF and ADB were nearer to the random guess (0.5) (Figure 2). The variability in the prediction probabilities was higher for the XGB methods as compared to others, where the probabilities were evenly distributed around the random values. For SVM, most of the probabilities were found to be higher than the random guess (0.5), where the mode of the distribution was found to be around 0.75 for all three datasets (Figure 2).

### 2.5. Analysis with Leave-One-Out Cross Validation

The performance of SVM was further analyzed by following a leave-one-out cross validation (LOOCV) approach. Similar to the 5-fold cross validation (5F-CV), higher accuracies were found with Pre-miRNA + miRNA as compared to the Pre-miRNA and miRNA datasets. For the miRNA dataset, the performance metrics of 5F-CV and LOOCV were observed to be almost same (Table 3). In contrast, the sensitivity of LOOCV was ~1% higher than that of 5F-CV but the specificity of LOOCV was ~1% less than that of 5F-CV (Table 3). When compared using the Pre-miRNA dataset, the sensitivity, F-score, and accuracy of 5F-CV were observed to be ~1% higher than the respective metrics of LOOCV, whereas the specificity and auROC of LOOCV were ~1% higher than that of 5F-CV (Table 3). The precision and auPRC of both 5F-CV and LOOCV were found to be almost same. In the case of the Pre-miRNA + miRNA dataset, the auROC and auPRC of LOOCV were higher as compared to that of 5F-CV, whereas the other performance metrics obtained with LOOCV and 5F-CV were similar (Table 3). The accuracies of both LOOCV and 5F-CV were found to be almost similar for all three datasets with few exceptions.

### 2.6. Prediction for the Independent Test Set

For the independent dataset, we collected 89 abiotic stress-responsive miRNAs from the existing studies [4,17,23,42,51,55,58,66,67,68,69,70]. After removing the identical sequences and the sequences with non-standard residues (other than A, U, G, C), 72 miRNAs were retained for the independent set. It was also ensured that these miRNAs were not present in the 1428 miRNAs collected for the training set. As far as the Pre-miRNA independent dataset is concerned, 70 Pre-miRNAs were retrieved for 70 miRNAs (out of 72) from the miRbase database. As far as a negative set is concerned, 100 sequences each for miRNAs and corresponding Pre-miRNAs were randomly taken from the miRBase database. Prediction for the test set was performed using the model trained with SVM with the selected feature sets. Summary of the test dataset and accuracies are given in Table 4. Similar to the 5-fold cross validation accuracies (Table 1), higher prediction accuracies were obtained for Pre-miRNA + miRNA as compared to the miRNA and Pre-miRNA test datasets. Specifically, overall accuracies of 62.33%, 64.85%, and 69.21% were obtained for the independent sets of miRNA, Pre-miRNA, and miRNA + Pre-miRNA, respectively (Table 4). Overall prediction accuracies of test instances of miRNA (62.33%), Pre-miRNA (64.85%), and miRNA + Pre-miRNA (69.21%) were also observed to be similar with the 5-fold cross validation accuracies of miRNA (65.33%), Pre-miRNA (66.40%), and miRNA + Pre-miRNA (71.40%), respectively.

### 2.7. Prediction Server ASRmiRNA

For easy implementation of the proposed approach, we established an online prediction server ASRmiRNA (http://cabgrid.res.in:8080/asrmirna, accessed on 28 December 2021) for prediction of abiotic stress-responsive miRNAs and Pre-miRNAs. The front end of the server was designed using HTML, whereas the developed R-code is run at the back end with the help of PHP. The prediction can be made by using three types of datasets, i.e., miRNA, Pre-miRNA, and miRNA + Pre-miRNA. The user has to paste the sequences in FASTA format in the text box provided. In the case of miRNA + Pre-miRNA, the user has to supply both miRNAs and their corresponding Pre-miRNAs in the respective text boxes. The results are presented in tabular format, where the probabilities with which each sequence is predicted as stress-responsive or non-stress-responsive are provided.

## 3. Discussion

Plants experience several environmental stresses that adversely affect their growth and development [71,72]. Abiotic stresses such as cold, drought, heat, light, oxidation, and salt are the major ones that limit the growth and productivity of crop plants to a large extent [73,74]. In defense of such abiotic stresses, plants adopt different mechanisms, and regulating the expression of abiotic stress-responsive genes via miRNAs is one of them. The miRNAs act as a post-transcriptional regulators of gene expression in a sequence-specific manner to respond to different abiotic stresses, where the gene expression is regulated via translational inhibition [50]. The miRNA recruits the Argonaute proteins to specifically target mRNA via base-pairing, to repress their translation and stability [75]. Although it cannot be guaranteed that the miRNA sequence is solely responsible for the abiotic stress response, at the same time it cannot be ignored that the whole process of translational repression occurs with the specific base pairing of miRNA with the target region where the order of nucleotides in the miRNA plays an important role. In particular, targeting is dependent upon the base pairing of the seed region, nucleotides (nts) 2–7 of the miRNA to sites in mRNA 3′UTRs. In addition, the 3′ end of the miRNAs has also been found to be involved in regulating the target specificity and regulation [76], where the extent of base-pairing to the miRNA 3′-end can influence the stability of the miRNA itself, and this signifies the importance of miRNA sequence beyond the seed region [75]. Thus, we believe the sequence of miRNA itself has a pivotal role in the whole process of regulation of the gene expression. Therefore, identification of abiotic stress-responsive miRNAs based on the sequence information is an important area of research as far as the plant response to different environmental stresses is concerned. The existing methods for miRNA identification such as cloning, high throughput sequencing, and microarrays [8] are costly as well as time consuming. Thus, there is a need to develop an alternate method for identification of abiotic stress-responsive miRNAs, and hence the present study is focused on developing a computational method for prediction of miRNAs associated with abiotic stress response. More specifically, we employed machine learning methods for prediction of abiotic stress-responsive miRNAs using sequence-derived features. For prediction, we employed three types of datasets, i.e., miRNA, Pre-miRNA, and combination of miRNA and Pre-miRNA.

One of the most important tasks in machine learning-based prediction using biological sequence data is to encode the sequences into numeric features, as machine learning algorithms (MLA) can only take numerical inputs [2,77,78,79,80]. Further, the miRNA sequences are only 20–24 nucleotides long, which is also a limitation to generate large number of discriminative features. In the present study, we utilized the psudo *K*-tuple nucleotide compositional (PseKNC) features to transform the miRNA sequences into numeric feature vectors. The PseKNC was successfully adopted in earlier studies [61,81,82,83] for prediction using biological sequence data. Here, we considered K = 2, 3, 4, and 5, and therefore total numbers of feature generated were $4^{2}+3+4^{3}+3+4^{4}+3+4^{5}+3=1372.$ Because miRNA sequences are only 20–24 nucleotides long, there is a higher probability of generated features containing large numbers of 0s, which may introduce redundancy in the feature set. 

Prediction accuracies can be misleading with the presence of redundant or irrelevant features. Thus, it is important to select important features among all the generated features. In this study, we utilized the SVM-RFE [84] for selecting the feature set for best discriminating the abiotic stress-responsive miRNAs and Pre-miRNAs from non-stress-responsive miRNAs and Pre-miRNAs, respectively. The SVM-RFE method has also been successfully adopted in many applications such as signal processing [85], genomics [86,87], proteomics [88], and metabolomics [89,90]. After ranking all the features using SVM-RFE, the classification accuracies in terms of auROC and auPRC were plotted in an iterative manner by adding 10 features at a time, until all the features are exhausted. The numbers of features were selected where the higher values of auROC and auPRC were obtained. The number of features selected for miRNA, Pre-miRNA, and Pre-miRNA + miRNA datasets were 200, 250, and 500, respectively. The number of features selected for Pre-miRNA dataset is larger than that of miRNA, because it is expected that the number of sparse features for miRNA will be more as compared to Pre-miRNA, as miRNA sequence length is much smaller than that of Pre-miRNA. Further, the number of features selected with the Pre-miRNA + miRNA dataset is larger and this because the number of features considered for Pre-miRNA + miRNA dataset is 2744 (1372 for miRNA and 1372 for Pre-miRNA).

Due to high generalized predictive ability, the SVM has been successfully utilized in different domains of research. Ability to handle large and noisy data is also one of the reasons for wide and successful implementation of SVM in many computational studies [91,92,93,94]. Thus, we preferred SVM over other machine learning algorithms for classification of stress responsive miRNAs and Pre-miRNAs. Further, the prediction accuracies were measured by following the 5-fold cross validation approach. The prediction accuracies with miRNA and Pre-miRNA datasets were found to be almost similar. This may be because 20–24 nucleotides long miRNA was present within Pre-miRNA, and also the number of features was almost the same. In contrast, the prediction accuracies with the Pre-miRNA + miRNA dataset were ~5% higher compared to those of the miRNA and Pre-miRNA datasets. One of the possible explanations for higher accuracy may be the use of larger number of selected features (500) in the case of the Pre-miRNA + miRNA dataset.

The performance of SVM was further compared with that of other start-of-the-art machine learning algorithms, i.e., random forest (RF), adaptive boosting (ADB), and extreme gradient boosting (XGB). The SVM outperformed all three learning algorithms. SVM achieved ~10% higher accuracy than the RF and 12–14% higher accuracy than that of the ADB and XGB algorithms. Further, RF was observed to achieve higher accuracy as compared to that of the ADB and XGB learning methods when miRNA and Pre-miRNA datasets were used. In contrast, similar accuracies were found for all three methods when predicting with the Pre-miRNA + miRNA dataset. The lower accuracies of prediction for RF, ADB, and XGB may be because the features selected using SVM may not be appropriate to achieve higher accuracy with the other learning methods. The higher accuracies of SVM as compared to RF, XGB, and ADB were also obtained in our earlier studies [95,96].

The performance of the proposed approach (SVM with selected features) was also assessed by using an independent dataset that comprises sequences of miRNAs collected from existing studies. The Pre-miRNA sequences of the respective miRNAs were obtained from the miRBase database. The overall accuracies for the independent set were found to be almost similar with the cross-validation accuracies. This shows that the accuracies were neither over estimated nor under estimated. Similar to 5-fold cross validation accuracies, the accuracies of Pre-miRNA + miRNA independent dataset were observed to be higher than those of miRNA and Pre-miRNA independent sets.

The 376 miRNA sequences used in this study were from 108 plant species with >50% sequences for *Arabidopsis thaliana*. In other words, each plant species has ~3 sequences on average. As it is difficult to train a machine learning model using fewer numbers of observations, the prediction was not performed on individual plant species.

Development of any computational method must be available in the form of a software package or prediction server for its usefulness by the user community, particularly those who are from a non-computational background. Thus, we established a prediction server ASRmiRNA (http://cabgrid.res.in:8080/asrmirna, accessed on 28 December 2021) for the identification of miRNAs and Pre-miRNAs associated with the abiotic stress response of plants.

## 4. Materials and Methods

### 4.1. Retrieval and Processing of Sequence Data

We collected 1428 abiotic stress-responsive miRNA sequences from the PncStress database [59], which is accessible at http://bis.zju.edu.cn/pncstress/, accessed on 28 December 2021. This database contains 4227 experimentally validated stress-responsive non-coding RNAs (miRNA, LncRNA, and circRNA) from 114 plants, covering 48 biotic and 91 abiotic stresses. After removing the identical sequences, we obtained 668 miRNA sequences. The abiotic-stress responsive miRNA sequences were used to construct the positive set. For the negative set, we collected 9716 miRNA sequences from the miRBase database [97], which is available at https://www.mirbase.org/, accessed on 28 December 2021. Out of 9716 sequences, 5701 sequences were retained after excluding the identical sequences, and these were utilized as the negative set. To avoid homologous bias in the prediction accuracy, both positive and negative datasets were subjected to the homology reduction at 80% sequence identity using the CD-HIT program [98]. After removing the redundant sequences, a total of 376 and 3823 miRNA sequences were respectively obtained for the positive and negative sets. In addition, we also used the Pre-miRNA sequences of the abiotic stress-responsive miRNAs for prediction. Out of 376 non-redundant miRNAs, we retrieved 251 corresponding Pre-miRNAs from the miRBase database. All the Pre-miRNAs were available for the 3823 miRNA sequences of the miRbase. Thus, we considered 251 abiotic stress-responsive Pre-miRNAs as the positive set and 3823 Pre-miRNAs as the negative set as far as prediction with Pre-miRNA sequences is concerned. Furthermore, another dataset was prepared by considering both miRNAs and Pre-miRNAs. In summary, three datasets were prepared for the analysis, i.e., miRNA, Pre-miRNA, and Pre-miRNA + miRNA. 

### 4.2. Pseudo K-Tuple Nucleotide Compositional Features

In this study, we generated pseudo *K*-tuple nucleotide compositional (PseKNC; Chen et al. [99]) features to transform each miRNA sequence into a numeric feature vector because the pseudo composition of nucleotides can take into account the long-range sequence order effect [99]. Each sequence can be transformed to a numeric vector of $4^{K}+\lambda$ elements, where a set of correlation factors captured the sequence order effect. Mathematically, the $j^{\mathrm{th}}$ tier correlation factor between all the $j^{\mathrm{th}}$ most contagious *K*-tuple nucleotides in a sequence of L nucleotides is

$$\delta_{j}=\frac{1}{L-K-j+1}\sum_{i=1}^{L-K-j+1}\emptyset_{i,\;\;i+j},\;j<L-K,$$

where

$$\begin{array}{c} \emptyset_{i,\;\;i+j}=\frac{1}{R}\sum_{r=1}^{R}{\left[\varphi_{n}\left(s_{i}s_{i+1}s_{i+2}\ldots s_{i+K-1}\right)-\varphi_{n}\left(s_{i+j}s_{i+j+1}s_{i+j+2}\ldots s_{i+j+K-1}\right)\right]}^{2},\\ i=1,\;2,\;\ldots ,\;L-K+1;j=1,\;2,\;\ldots ,\;\lambda ;\;\lambda <L-K\;and\;s_{i}\in \left\{A,\;U,\;G,\;C\right\}. \end{array}$$

Here, $\varphi_{n}\left(s_{i}s_{i+1}s_{i+2}\ldots s_{i+K-1}\right)$ is the $r^{\mathrm{th}}$ property of the *K*-tuple nucleotides $s_{i}s_{i+1}s_{i+2}\ldots s_{i+K-1}$ in the sequence, and R is the total number of such property [99]. Now, the $4^{K}+\lambda$ dimensional feature vector can be represented as $\vartheta_{1},\;\vartheta_{2},\;\ldots ,\;\vartheta_{4^{k}},\;\vartheta_{4^{k}+1},\;\ldots ,\;\vartheta_{4^{k}+\lambda }$, where

$$\vartheta_{m}=\{\begin{array}{c} \frac{g_{m}^{K-tuple}}{\sum_{i=1}^{4^{K}}g_{i}^{K-tuple}+\omega \sum_{j=1}^{\lambda }\delta_{j}},\;\;\;\;\;\;\;\;\;\;\;\;\;\;\;\;\;\;\;\;\;\;\;\;\;\;\;1\leq m\leq 4^{K}\\ \frac{\omega \delta_{m-4^{K}}}{\sum_{i=1}^{4^{K}}g_{i}^{K-tuple}+\omega \sum_{j=1}^{\lambda }\delta_{j}},\;\;4^{K}+1\leq m\leq 4^{K}+\lambda \end{array}.$$

Here, $g_{m}^{K-tuple}$ is the normalized frequency of the $i^{\mathrm{th}}$ *K*-tuple nucleotides in the sequence, and ω is the weight factor. The first $4^{K}$ elements reflect the effect of *K*-tuple nucleotide composition and $4^{K}+1$ to $4^{K}+\lambda$ elements reflect the effect of long-range sequence order. As the miRNA sequences are around 20–24 nucleotides, we considered up to 3-tier correlation only, hence *λ* = 3 was taken. The weight factor ω=0.2 w as considered. Specifically, the values of *K* = 2, 3, 4, and 5 were considered and the number of features generated were 19, 67, 259, and 1027, respectively. In total, 1372 features were generated for each miRNA sequence. The Pse-in-One web server [100] was utilized to generate the PseKNC features.

### 4.3. Prediction with Support Vector Machine Algorithm

We used support vector machine (SVM; Coretes and Vapnik [101]) for prediction, as it has been successfully employed for prediction in existing biological studies [78,79,102,103,104,105,106]. The SVM maps the input data to high dimensional features and searches for an optimal separating hyper plane with maximum margin for the classification of observations of different classes. Let $y_{i}\in \left\{-1,\;1\right\}$ be the class label for the $i^{\mathrm{th}}$ observation vector $x_{i}\;\left(i=1,\;2,\;\ldots ,\;N\right)$. The hyper plane can then be written as $w^{\prime }x+b=0$, where w and b represent weight and bias factors, respectively. The process of choosing the optimal hyper plane involves a convex optimization problem, which can be formulated as

$$minimize\;\;\frac{1}{2}{\left\|w\right\|}^{2}+C\overset{N}{\underset{i=1}{\sum }}\varphi_{i}\;$$

subject to the constraints

$$y_{i}(w^{\prime }x+b)+\varphi_{i}\geq 1\;\mathrm{and}\;\varphi_{i}\geq 0;i=1,\;2,\;\ldots ,\;N\;$$

The cost parameter C controls the trade-off between the margin and the misclassification error, and $\varphi_{i}$ is the slack variable represents the distance between the boundary and the classification point. The minimization can also be formulated as a maximization problem with Lagrangian theory, i.e.,

$$maximize\;\;\overset{N}{\underset{i=1}{\sum }}\alpha_{i}-\frac{1}{2}\overset{N}{\underset{i=1}{\sum }}\overset{N}{\underset{j=1}{\sum }}\alpha_{i}\alpha_{j}y_{i}y_{j}K\left(x_{i},x_{j}\right)\;$$

subject to the constraints

$$\sum_{i=1}^{N}\alpha_{i}y_{i}=0\;\mathrm{and}\;0\leq \alpha_{i}\leq C;\;i=1,\;2,\;\ldots ,\;N$$

Here, α is the Lagrangian multiplier and K is the kernel function that plays the most important role of transforming the input dataset to high-dimensional feature space in which the observations of different classes are linearly separable. As far as kernel function K is concerned, we employed the radial basis (RBF) kernel function that can be represented as $K\left(x_{i},x_{j}\right)=exp\left(\|x_{i}-x_{j}\|{}^{2}\right)$. The decision function can now be written as $f\left(x\right)=sign\left(\overset{N}{\underset{i=1}{\sum }}\alpha_{i}y_{i}K\left(x,x_{i}\right)\right)$, where f(x)>0 indicates that the observation vector x is classified in the +1 class and −1 class otherwise. The SVM was implemented using the “e1071” R-package [107].

### 4.4. Feature Selection with SVM-Recursive Feature Elimination Approach

Feature selection helps to filter out the redundant and noisy features and thereby reduce the computational complexity and improve the classification accuracy [108,109]. In this study, we employed the SVM-recursive feature elimination (SVM-RFE; Guyon et al. [110]) method for selection of important features. The SVM-RFE is a backward feature elimination technique, where the features are removed iteratively based on SVM classifier weights. The weight vector for the SVM classifier can be obtained as $w=\sum_{i=1}^{P}\alpha_{i}y_{i}x_{i}$, where P is the total number of features, $\alpha_{i}$ is the Lagrangian multiplier estimated from the training dataset, $x_{i}$ is the feature vector for the *i*^th^ observation, and $y_{i}$ is the class label of the corresponding observation. The top-ranked features that are removed in the last iteration of SVM-RFE are considered most important, whereas the bottom-ranked ones are the least informative and removed in the first iteration. In other words, an SVM model is built in each iteration based on the current features subset F, and the weight (w) of each feature in F is computed. The features are then ranked on the basis of $w^{2}$ and the bottom-ranked features are removed from F. This procedure is repeated until F is empty. For a specific application, it is important to determine how many features should be retained for the analysis. In this study, the top features that induced a classifier with best classification accuracy were selected as per earlier studies [90,110]. The SVM-RFE method was implemented using the “sigFeature” R-package [111].

### 4.5. Cross-Validation Approach

**Figure 3:**
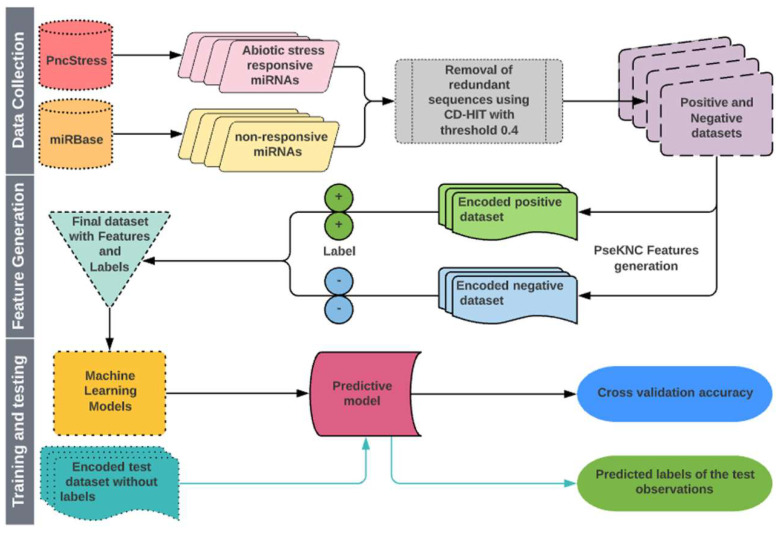
Flow diagram showing the steps involved in the proposed approach for prediction of abiotic stress-responsive Pre-miRNAs and miRNAs.

Cross-validation (CV) procedure is a widely accepted approach to estimate the accuracy of classification/prediction algorithms by running training and testing on different partitions of the dataset [112,113]. The K-fold and leave-one-out CV (LOOCV) are often used to evaluate the performance of learning algorithms. In this study, we used both 5-fold CV and LOOCV procedures to evaluate the performance of classifiers. For 5-fold CV, both positive and negative datasets were randomly partitioned into five equal-size subsets. One subset each from positive and negative sets constituted the test set that was used to evaluate the model trained with the remaining four subsets from both classes. Each subset was tested exactly once, and the process was repeated five times. The performance was calculated by taking the average over all five subsets. The LOOCV is the least arbitrary method, as it always yields a unique result for a given dataset [104]. In LOOCV, each observation was singled out as a test instance and the remaining observations were used to train the model. This process was repeated until all the instances were exhausted as test instances. A flow chart describing all the steps involved in the proposed approach is shown in Figure 3.

### 4.6. Performance Evaluation Criteria

To objectively evaluate the anticipated accuracy of the classifiers, a set of quantitative performance metrics such as sensitivity (*Sen*), specificity (*Spe*), accuracy (*Acc*), precision (*Pre*), and *F-score* was utilized. The metrics are defined as follows:

$$Sen=\frac{TP}{TP+FN}$$

$$Spe=\frac{TN}{TN+FP}$$

$$Acc=\frac{1}{2}\left(Sen+Spe\right)$$

$$Pre=\frac{TP}{TP+FP}$$

$$F-score=\frac{2\times TP}{2TP+FP+FN}$$

*TP*, *TN*, *FP,* and *FN* represent the number of true positives, true negatives, false positives, and false negatives, respectively. In addition, we also used area under receiver characteristic curve (auROC; Fawcett [114]) and area under precision-recall curve (auPRC; Davis and Goadrich [115]) to measure the performance of the classifiers.

### 4.7. Prediction with Balanced Dataset

The numbers of sequences in negative datasets (3823) were much larger than that of positive datasets—that is, miRNA (376), Pre-miRNA (251), and miRNA + Pre-miRNA (251). Prediction with such imbalanced datasets may result in higher accuracy for the negative class as compared to the positive class [61,78,79,116,117]. To avoid such biasness while predicting with MLA, balanced datasets containing equal number of instances from both positive and negative classes were prepared, where the sequences of the negative class were randomly drawn from the available sequences. For instance, the balanced dataset for miRNA contains 376 positive and 376 negative sequences (randomly drawn from 3823 sequences). The balanced datasets for Pre-miRNA and miRNA + Pre-miRNA were similarly prepared.

## Figures and Tables

**Table 1:** Performance metrics of support vector machine (SVM) for predicting abiotic stress associated miRNAs and Pre-mirNAs. The predictions were made with the selected feature sets. The prediction accuracies with the Pre-miRNA + miRNA dataset were found higher as compared to that of the miRNA and Pre-miRNA datasets.

Dataset	Sen (%)	Spe (%)	Acc (%)	Pre (%)	F-Score (%)	auROC (%)	auPRC (%)
miRNA	66.13	64.53	65.33	65.09	65.61	70.21	69.96
Pre-miRNA	69.20	63.60	66.40	65.53	67.31	69.71	65.64
Pre-miRNA + miRNA	74.00	68.80	71.40	70.34	72.12	77.94	77.32

**Table 2:** Performance metrics of random forest (RF), adaptive boosting (ADB), and extreme gradient boosting (XGB) methods. The performance of RF, ADB, and XGB were analyzed using the selected feature sets for predicting abiotic stress responsive miRNAs and Pre-miRNAs. The RF method achieved higher accuracies as compared to the other two methods. Nevertheless, the accuracies were not found to be much different among the three learning methods. For all three learning methods, the accuracies are observed to be higher with the Pre-miRNA + miRNA dataset.

Dataset	Method	Sen (%)	Spe (%)	Acc (%)	Pre (%)	F-Score (%)	auROC (%)	auPRC (%)
miRNA	RF	55.20	58.13	56.66	56.86	56.02	58.88	58.25
	XGB	51.21	56.00	53.61	53.78	52.46	54.79	56.03
	ADB	52.26	57.06	54.67	54.91	53.55	57.45	57.01
PremiRNA	RF	65.60	58.50	62.20	61.42	63.44	64.25	58.03
	XGB	55.61	56.40	56.00	56.04	55.82	58.26	54.91
	ADB	58.01	60.00	59.00	59.18	58.58	62.28	57.86
Pre-miRNA + miRNA	RF	63.20	62.00	62.60	62.45	62.82	64.63	60.28
	XGB	62.20	61.60	62.00	61.90	62.15	62.56	59.64
	ADB	61.60	59.60	60.60	60.39	60.99	63.55	59.96

**Table 3:** Prediction accuracies with 5-fold cross validation (5F-CV) and leave-one-out cross validation (LOOCV). The prediction accuracies are higher for the Pre-miRNA + miRNA as compared to the other two datasets. The performances with 5F-CV and LOOCV are similar when all the metrics are accounted for. Among the metrics, the auROC and auPRC are higher.

Dataset	Cross-Validation	Sen (%)	Spe (%)	Acc (%)	Pre (%)	F-Score (%)	auROC (%)	auPRC (%)
miRNA	5-Fold	66.13	64.53	65.33	65.09	65.61	70.21	69.96
	Leave-One-Out	67.02	63.56	65.29	64.78	65.88	70.28	70.17
Pre-miRNA	5-Fold	69.20	63.60	66.40	65.53	67.31	69.71	65.64
	Leave-One-Out	66.93	64.54	65.74	65.37	66.14	70.52	65.48
miRNA + Pre-miRNA	5-Fold	74.00	68.80	71.40	70.34	72.12	77.94	77.32
	Leave-One-Out	74.50	68.53	71.51	70.30	72.34	79.35	78.73

**Table 4:** Summary of the independent datasets and their prediction accuracies. The accuracies are on par with the accuracies of 5-fold cross validation. The accuracies are also observed higher for the miRNA + Pre-miRNA dataset.

Dataset	#Sequences		Performance Metrics		
	Positive	Negative	Sensitivity (%)	Specificity (%)	Accuracy (%)
miRNA	72	100	66.66	58.00	62.33
Pre-miRNA	70	100	65.71	64.00	64.85
miRNA + Pre-miRNA	70	100	71.42	67.00	69.21

## Data Availability

The data presented in this study are available at http://cabgrid.res.in:8080/asrmirna/dataset.html, accessed on 28 December 2021.
